# Thymoquinone Attenuates NF-κβ Signalling Activation in Retinal Pigment Epithelium Cells Under AMD-Mimicking Conditions

**DOI:** 10.3390/ijms262311473

**Published:** 2025-11-27

**Authors:** Nur Musfirah Mahmud, Luminita Paraoan, Tengku Ain Kamalden

**Affiliations:** 1 UM Eye Research Centre, Department of Ophthalmology, Faculty of Medicine, Universiti Malaya, Kuala Lumpur 50603, Malaysia; musfirah@um.edu.my; 2 Department of Life Sciences, Faculty of Science and Engineering, Manchester Metropolitan University, Chester Street, Manchester M1 5GD, UK; l.paraoan@mmu.ac.uk

**Keywords:** thymoquinone, oxidative stress, TNFα, retinal pigment epithelium, age-related macular degeneration

## Abstract

Oxidative stress in retinal pigment epithelium (RPE) cells plays a key role in the development of age-related macular degeneration (AMD), a leading cause of vision loss in the elderly. Thymoquinone, a bioactive antioxidant from Nigella sativa, has shown promise in reducing cellular oxidative stress. In AMD, prolonged exposure to oxidative stress may activate the NF-κβ signalling pathway in RPE cells, contributing to chronic inflammation, and its regulation by thymoquinone remains understudied. This study investigates the effects of thymoquinone in TNFα-induced RPE cells exposed to AGEs to mimic ageing conditions relevant for AMD. Gene and protein expression levels of NF-κβ pathway markers (P65, pP65 and Iκβα) were measured using qPCR and Western blotting, and statistical analysis was performed using Student’s *t*-test and one-way ANOVA. Thymoquinone pretreatment at 0.1 µM and 10 µM significantly reduced the expression of these markers in TNFα-stimulated RPE cells. Notably, AGE-exposed cells demonstrated a heightened response to thymoquinone compared to non-AGE-exposed controls. These findings suggest that thymoquinone modulates NF-κβ signalling and may serve as a potential adjuvant therapeutic agent for AMD.

## 1. Introduction

Age-related macular degeneration (AMD) is a complex disease prominently affecting the elderly aged 55 and above [1,2]. According to the American Academy of Ophthalmology, the prevalence of AMD in Asians is comparable to that in Caucasians—6.8% in the early stage and 0.56% in the late stage [3]. External and internal factors, including smoking, light exposure, nutrient intake, stress, sex, ethnicity, genetics, inflammation and oxidative stress, can increase the likelihood of getting AMD [4]. Excessive exposure to these factors may result in impairment and dysfunction of retinal pigment epithelium (RPE), photoreceptors, Bruch’s membrane and choroid, subsequently leading to visual loss, as seen in advanced stages of AMD [5,6].

Excessive buildup of oxidative stress within cells can lead to damage of key biomolecules, including proteins, lipids and nucleic acids [7]. The RPE is particularly susceptible to oxidative damage, owing to high metabolic activity, substantial oxygen consumption, continuous light exposure and its abundance of polyunsaturated fatty acids [8]. Moreover, intracellular oxidative stress generation in the RPE itself is also high due to high mitochondria content to provide adequate ATP for its physiological functions [9]. Oxidative stress accumulation in the RPE promotes the buildup of lipid byproducts, including lipofuscin and drusens, which represent key pathological hallmarks of AMD [10]. The death and impaired function of the RPE are the key drivers in AMD initiation and progression, whereas the photoreceptor and choroidal complex experience a secondary effect due to these changes, which leads to vision loss [11,12]. This explains the importance of tackling the mechanism behind RPE dysfunction to prevent AMD.

In AMD, the accumulation of oxidative stressors such as TNFα and advanced glycation end products (AGEs) is higher compared to normal ageing [13,14]. Both TNFα and AGEs have been shown to upregulate growth factors, including transforming growth factor β and the platelet-derived growth factor, which are critically involved in driving the pathological growth of new of choroidal vessels, a characteristic sign of wet AMD [15,16,17,18]. Additionally, both TNFα and AGEs have been reported to activate the NF-κβ signalling pathway, which also has a strong correlation with inflammation, implicating the pathogenesis of AMD [18,19,20,21]. Previously, AGE-exposed RPE cells have been shown to exhibit an augmented response towards TNFα stimulation through activation of NF-κβ signalling [22]. Genome analysis revealed that the NFκβ1 gene is one of 21 genes identified to have a high correlation with AMD, particularly present in retinal tissue [23]. Activation of the NF-κβ signalling pathway in the RPE has been shown to increase oxidative stress-related cytokines such as IL-6 and IL-8 [24]. Although the RPE has the antioxidative capacity to defend itself against such oxidative damage, it decreases with ageing [25]. These pathways are believed to influence cellular survival mechanisms, thereby reducing RPE cell death.

**Figure 1:**
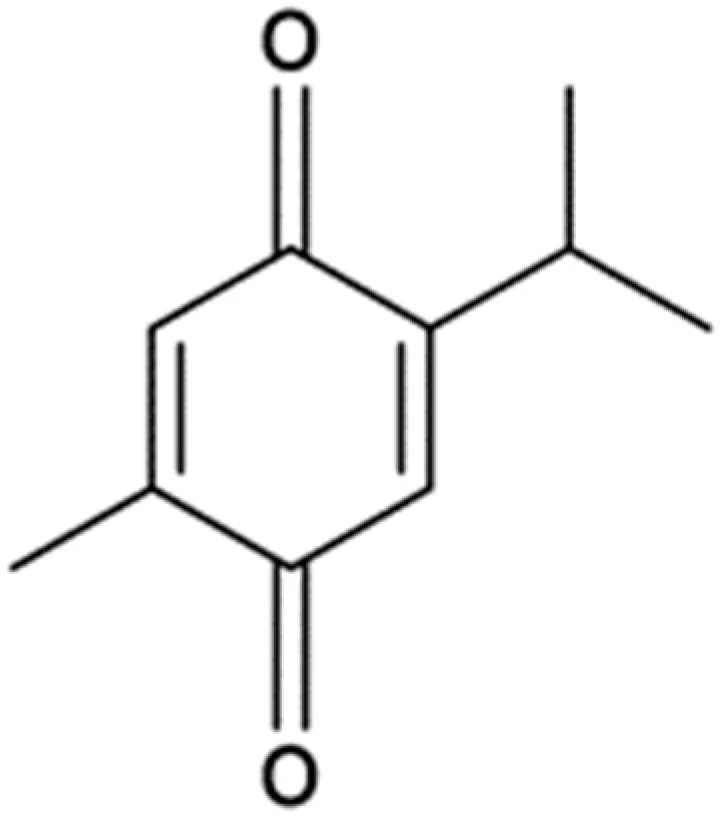
Chemical structure of thymoquinone.

Several studies have shown that naturally occurring antioxidants can reduce oxidative stress levels in cells and disease models. Chemical classes such as polyphenols (e.g., curcumin and resveratrol) [26] and flavonoids (e.g., epigallocatechin gallate from green tea) [27] have shown potential in delaying or protecting AMD through modulation of oxidative stress and inflammatory pathways. Thymoquinone (Figure 1), a bioactive compound classified as a monoterpene, shares some mechanistic pathways with these compounds, such as NF-κB signalling, supporting its potential role in AMD prevention [28]. This natural compound can be found in very few plants, including beebalms and species of herbs like thyme. However, research has identified thymoquinone as a primary constituent of black seed (*Nigella sativa*), which [29] has been used for wound healing [30], as well as its wide variety of health benefits for diseases including asthma [31], degenerative diseases [32,33], and inflammation-related diseases [34]. Despite the known health benefits of thymoquinone and its antioxidative effects, research into the therapeutic benefits of thymoquinone in oxidative stress-related eye diseases is very limited. Published studies have shown that thymoquinone exerts its effects via several key oxidative stress pathways, such as the NF-κβ signalling pathway, which is also implicated in AMD. This implies the potential protective or beneficial effects of thymoquinone in AMD and its potential to be considered in clinical practice. This study addresses the research gap with respect to the effects of thymoquinone on the oxidative stress pathway in human RPE cells exposed to AGEs mimicking AMD conditions.

## 2. Results

### 2.1. Dose–Response Effect of Thymoquinone on ARPE-19 Cells

**Figure 2:**
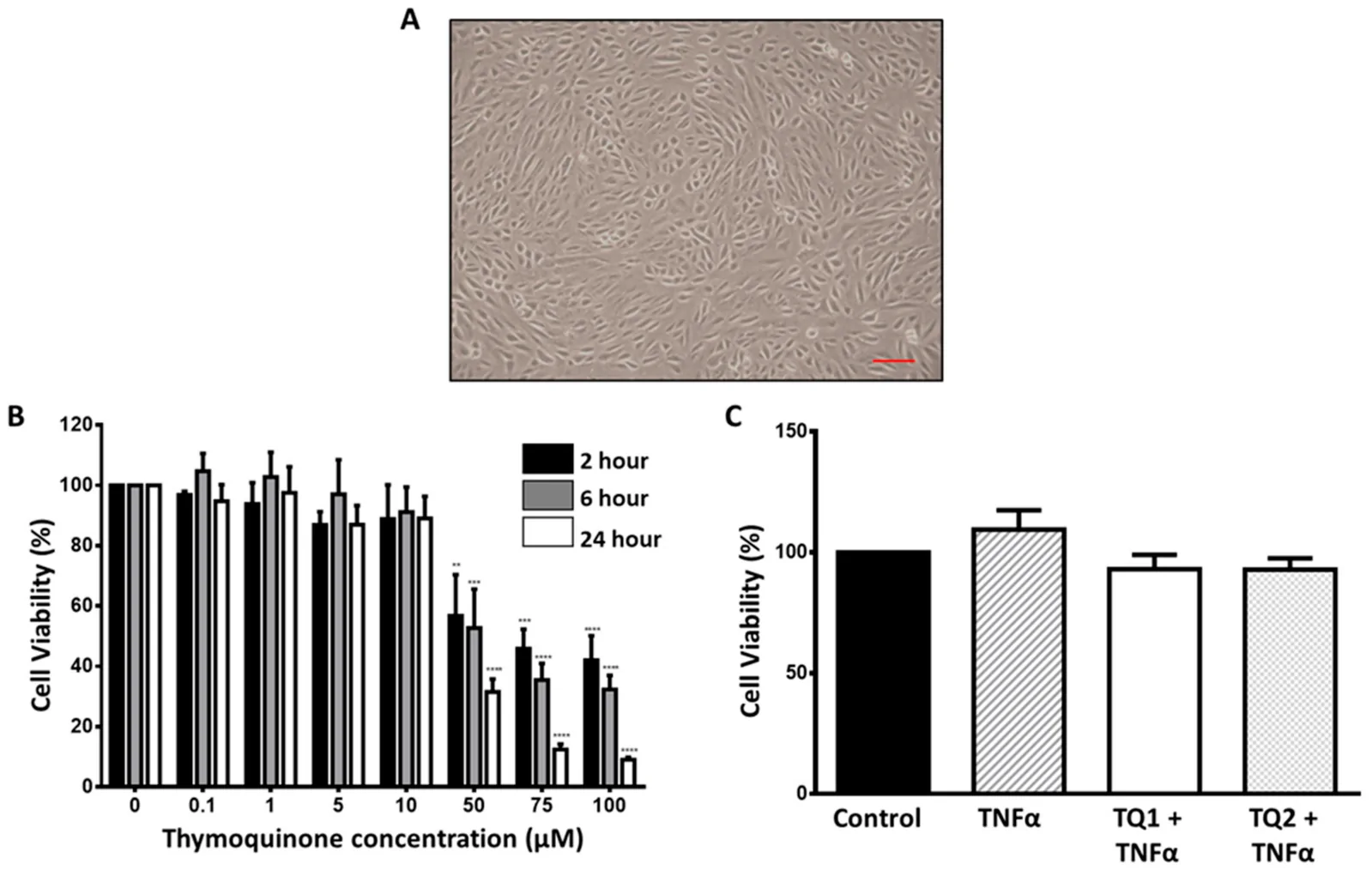
Effect of thymoquinone on ARPE-19 cells’ viability. (**A**) Representative image of ARPE-19 morphology by phase-contrast microscopy treated with thymoquinone; scale bar represents 100 µm. (**B**) ARPE-19 cells were treated with thymoquinone in the range of 0.1–100 µM for 2, 6 and 24 h, and cell viability was subsequently determined using an MTT assay; the graph represents average cell viability (mean ± SEM values, *n* = 3; Two-way ANOVA, ** *p* ≤ 0.01 vs. control, *** *p* ≤ 0.001 vs. control, **** *p* ≤ 0.0001 vs. control). (**C**) ARPE-19 cells were treated with a 10 ng/mL TNFα concentration for 2 h, after which cell viability was measured using an MTT assay; the graph represents average cell viability (mean ± SEM values, *n* = 3; Student’s *t*-test).

ARPE-19 cells were treated with thymoquinone in a dose- and time-dependent manner, with concentrations ranging from 0.1 µM to 100 µm at three different time points (2, 6 and 24 h). The findings indicate that thymoquinone concentrations up to 10 µM did not induce observable changes in the cell morphology (Figure 2A) nor viability of ARPE-19 cells (Figure 2B, Table S1). Therefore, two concentrations—the highest (10 µM) and the lowest (0.1 µM) concentration of thymoquinone at the 6 h exposure period—were selected for subsequent analyses. Additionally, we showed that both treatments—thymoquinone and TNFα together at the determined concentrations—were non-lethal to the cultured ARPE-19 cells (Figure 2C, Table S2).

### 2.2. Effect of Thymoquinone Against TNFα-Induced Oxidative Stress in Ageing ARPE-19 Cells via Modulation of the NF-κβ Pathway

**Figure 3:**
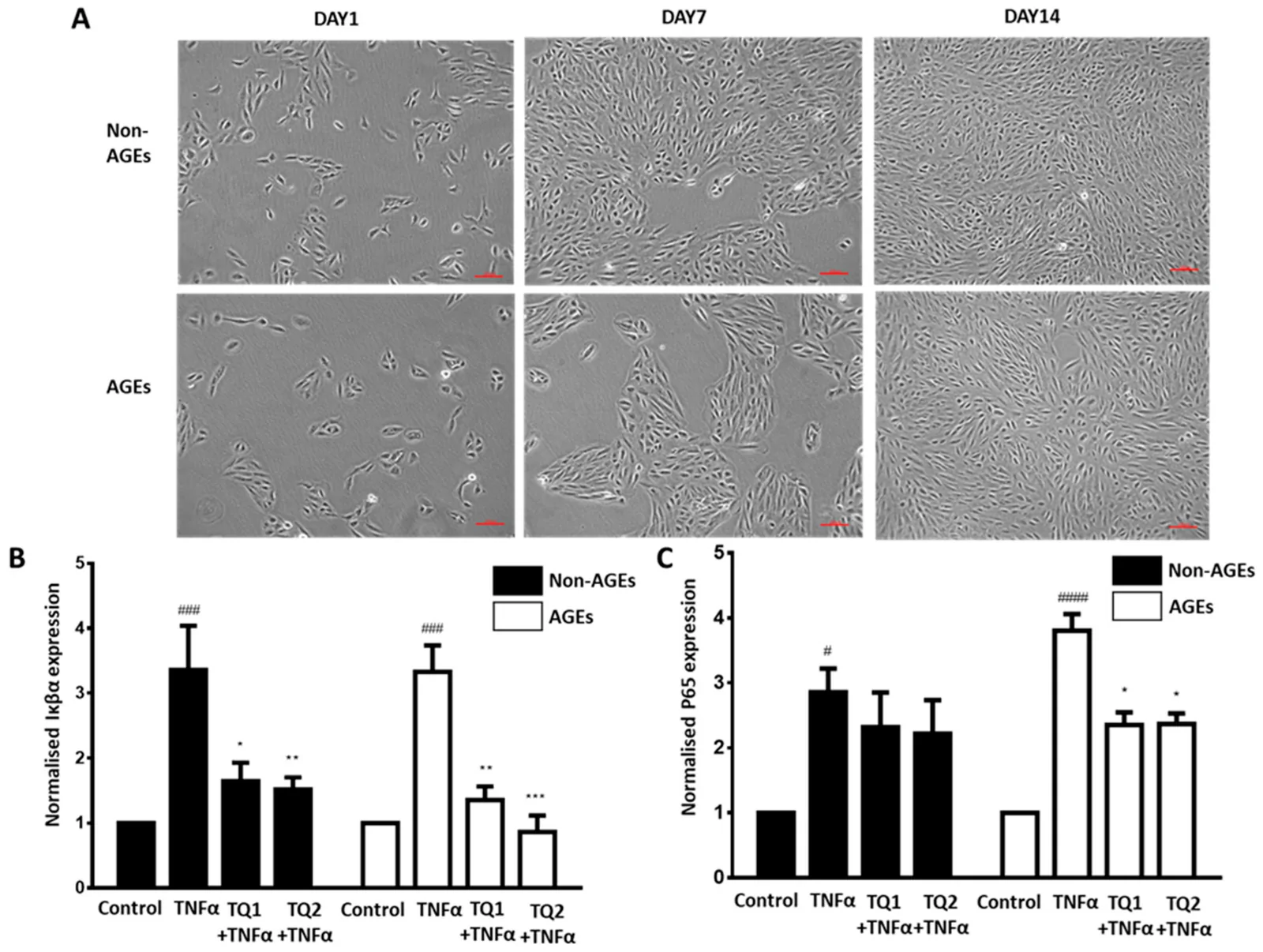
Protective effect of thymoquinone against TNFα-induced oxidative stress in ARPE-19 cells. ARPE-19 cells were pretreated with thymoquinone (TQ1: 0.1 μM; TQ2: 10 μM) for 6 h, followed by TNFα treatment (10 ng/mL) for 2 h. (**A**) Morphology of ARPE-19 cells by phase-contrast microscopy cultured on non-modified Matrigel (Non-AGEs) and AGE-modified Matrigel (AGEs) for 14 days; scale bar represents 100 µM. (**B**,**C**) Relative mRNA expression of P65 and Iκβα in ARPE-19 cells treated with thymoquinone and TNFα normalized to β-actin and β-tubulin analysed by qPCR; data are presented as mean ± SEM values, *n* ≥ 2; one-way ANOVA, # *p* ≤ 0.05 vs. control, ### *p* ≤ 0.001 vs. control, #### *p* ≤ 0.0001 vs. control, * *p* ≤ 0.05 vs. TNFα treated, ** *p* ≤ 0.01 vs. TNFα treated, *** *p* ≤ 0.001 vs. TNFα-treated.

The activation of the NF-κβ pathway of the NF-κB signalling cascade is a key driver of inflammation contributing to the development and progression of AMD. We sought to understand the effect of thymoquinone against TNFα-induced oxidative stress in ARPE-19 cells via the NF-κβ pathway. ARPE-19 cells were cultured on AGE-exposed Matrigel to mimic AMD conditions in vitro. We looked into the effect of thymoquinone on the NF-κβ signalling pathway in the AMD model of ARPE-19 cells to determine if thymoquinone is able to decrease the activation of the NF-κβ signalling pathway in ARPE-19 cells and, hence, reduce inflammatory reactions. ARPE-19 cells were cultured for two weeks in culture medium and pretreated with two different concentrations of thymoquinone (TQ1 = 0.1 μM and TQ2 = 10 μM) for 6 h prior to the stimulation of TNFα (10 ng/mL) for the specified time interval to investigate whether thymoquinone could reduce the activation of the NF-κβ signalling pathway. Figure 3A shows the AGE-exposed and non-AGE-exposed ARPE-19 cells after 2 weeks in culture. The growth of AGE-exposed cells is slower compared to non-AGE-exposed cells; however, by day 14, the cell growth is comparable between the two groups. The alteration of the NF-κβ signalling pathway was measured by detecting the expression of its key effector genes (P65 and Iκβα) and proteins (P65, pP65 and Iκβα) through quantitative PCR and Western blotting, respectively. As illustrated in in Figure 3B,C (Tables S3 and S4), the gene expression of P65 and Iκβα is significantly increased when treated with TNFα and significantly lowered with thymoquinone pretreatment.

**Figure 4:**
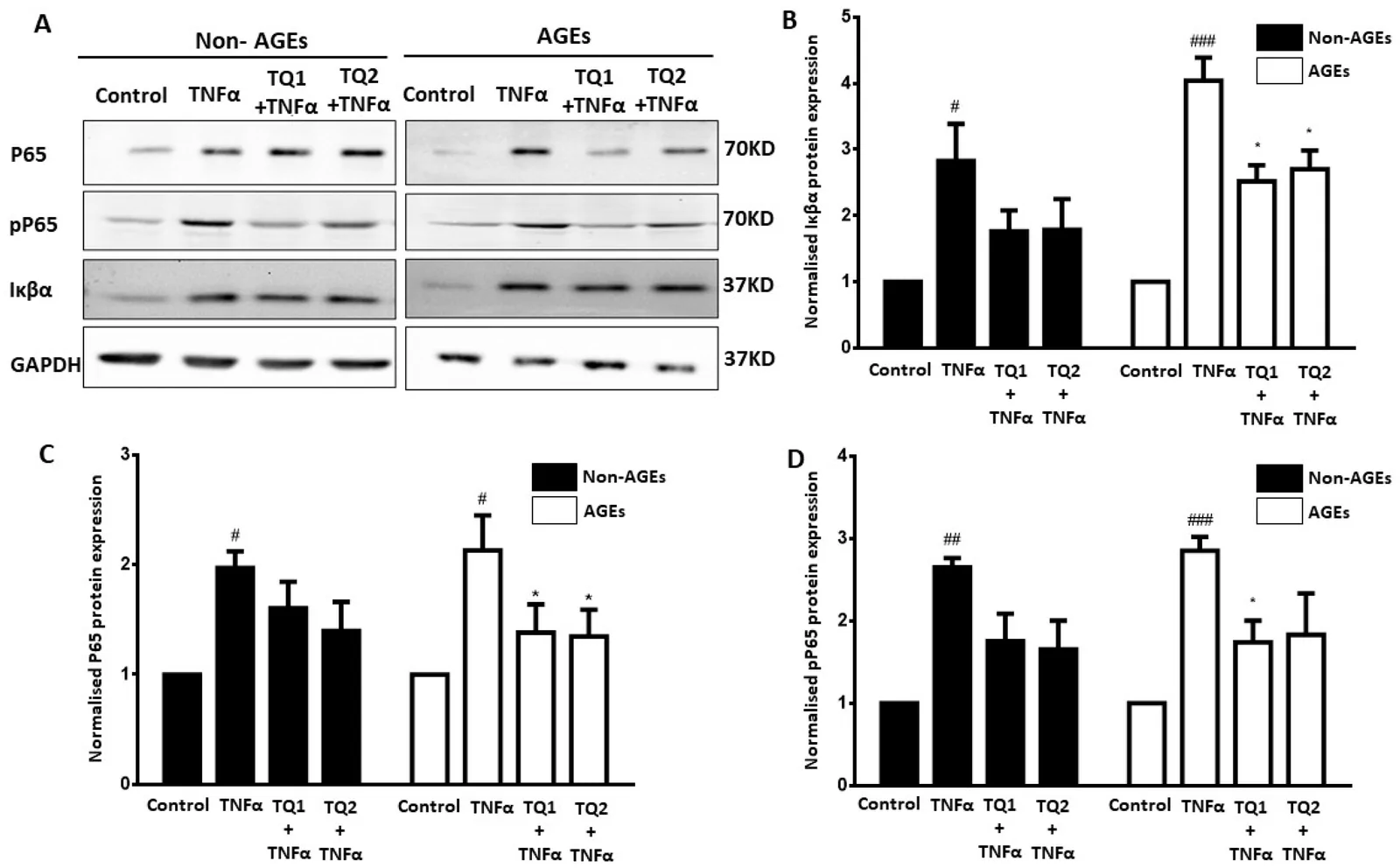
Protective effect of thymoquinone against TNFα-induced oxidative stress in ARPE-19 cells. ARPE-19 cells were pretreated with thymoquinone (TQ1: 0.1 μM; TQ2: 10 μM) for 6 h, followed by TNFα treatment (10 ng/mL) for 2 h. (**A**) Representative blot image of non-AGE-exposed and AGE-exposed ARPE-19 samples probed with anti-P65, anti-Iκβα and anti-phosphorylated p65 with GAPDH as a loading control; (**B**–**D**) Average normalized protein expression (Iκβα, P65 and pP65) in ARPE-19 cells treated with thymoquinone and TNFα normalized to GAPDH; data are presented as mean ± SEM values, *n* = 2; one-way ANOVA, # *p* ≤ 0.05 vs. control, ## *p* ≤ 0.01 vs. control, ### *p* ≤ 0.001 vs. control, * *p* ≤ 0.05 vs. TNFα-treated.

In addition, a similar trend was observed at protein expression levels of P65, pP65 and Iκβα (Figure 4A–C, Tables S5–S7). Pretreatment with thymoquinone at both concentrations was able to reduce significant increases in P65, pP65 and Iκβα, both at mRNA expression and protein expression levels. Importantly, in the AGE-exposed cells, the levels of both genes and proteins were significantly lowered by pretreatment with thymoquinone. Though the non-AGE-exposed cells showed the same decreasing trend in the levels of P65, pP65 and Iκβα protein expression, the changes were not statistically significant. Therefore, we speculated that thymoquinone was able to suppress TNFα-induced NF-κβ signalling activation in AGE-exposed RPE cells by modulating the levels of P65, pP65 and Iκβα in the cells.

## 3. Discussion

Thymoquinone has been shown to possess strong antioxidant activity, providing cellular protection against oxidative damage and apoptosis [35,36,37]. Inflammation-related oxidants such as malondehyde, IFN-γ, TNF-α, COX-2, IL-6, IL-8, IL-16 and IL-12A were shown to be significantly reduced by pretreatment with thymoquinone [35,38,39]. With the important role of NF-κβ signalling as an inflammatory pathway, thymoquinone may act by altering the key NF-κβ effectors. Several studies have shown an association between NF-κβ and thymoquinone [40,41,42]. TNFα, which is a known oxidant, plays an important role in inflammation-related pathways and is upregulated in the case of AMD [43,44]. In addition, TNFα has been shown to trigger activation of the pro-inflammatory NF-κβ signalling pathway [20]. Therefore, this study investigated the effects of thymoquinone on the TNFα-induced NF-κβ signalling pathway in ARPE-19 cells as a cellular model of aged RPE cells simulating AMD. At the cellular level, our findings demonstrate that thymoquinone has an antioxidative capacity in RPE cells by modulating key effectors of the NF-κβ signalling pathway, as evidenced by attenuation in levels of P65, pP65 and Iκβα expression.

The involvement of inflammation in AMD has been well established, and TNFα plays an important role in promoting inflammatory response imbalance, contributing to AMD pathogenesis [45]. TNFα expression is significantly higher in eyes with neovascular AMD compared to those from normally aged individuals [43,44]. In addition, TNFα is a potent pro-inflammatory agent in human RPE cells through NF-κβ signalling, a signalling pathway that modulates several inflammation-related genes such as interleukin-1β and interleukin 18 [20]. In this study, 10 ng/mL TNFα was used to challenge NF-κβ activation in ARPE-19 cells, and this concentration was shown to be non-lethal to the cells (Figure 2C), proving that the result we observed in these experiments is a reaction to cellular response. NF-κβ is a transcription factor found inactive in the cytoplasm. In response to stimuli, the NF-κβ translocates into the nucleus to initiate the transcriptional activation of various pro-inflammatory-related genes [46]. NF-κβ activity was markedly higher in the RPE cells derived from the AMD model compared to controls [47]. Previous findings have shown the upregulation of key effectors of NF-κβ, including P65, pP65 and Iκβα, where RPE cells have an augmented response to TNFα treatment [22].

Studies have suggested that antioxidant dietary intakes help reduce the risk of AMD. Antioxidants such as lutein [48], curcumin [49], fisetin [50], luteolin [50], quercetin [51] and astaxanthin [52] have been tested and reported to provide a protective mechanism against oxidative stress in the RPE. However, not many studies have reported the effect of thymoquinone on the RPE. Previous studies in the eye using thymoquinone focused on dry eye [53], conjunctivitis [54], glaucoma [55] and diabetic retinopathy [37]. These in vivo studies showed that thymoquinone exhibited protective effects by reducing the secretion of interleukins (1L-4, IL-5 and IL-13) and decreased the elevation of oxidants such as malondialdehyde, nitric oxide and TNF-α, as well as caspase-3 activity. In addition, thymoquinone was found to exert its effects through modulation of NF-κβ signalling [56]. Therefore, with the increased susceptibility of the ageing RPE towards oxidative stress and inflammation via NF-κβ signalling, we aimed to investigate the effect of thymoquinone against NF-κβ signalling activation in ARPE-19 cells as a model of AMD. In our recent review, we discussed different possible pathways in which thymoquinone may act in the retina [57]. Studies have highlighted thymoquinone as a promising bioactive molecules with therapeutic potential for neurodegenerative diseases such as Alzheimer’s disease (AD) and Parkinson’s disease (PD) [36,58,59]. AD and PD have been discovered to share nearly the same disease mechanism as AMD, characterized by age-associated increases in oxidative stress and inflammatory pathway activation [9,60,61]. In experimental models of AD and PD, thymoquinone has demonstrated neuroprotective actions through multiple mechanisms: modulation of oxidative stress balance, suppression of AB plaque formation, preservation of mitochondrial structure and functions and regulation of the level of anti-inflammatory and pro-inflammatory genes, resulting in neuronal cell death [59,62,63,64]. Based on this evidence, we postulated that thymoquinone may also be useful in the treatment of AMD, as an ocular neurodegeneration disease.

Thymoquinone has a wide safety profile of up to 2400 mg/kg in rodents and up to 200 mg/day in adults, depending on the route of administration [65,66,67]. In vitro, we showed that concentrations up to 10 µM for 6 h did not affect cell growth. Two concentrations of thymoquinone (0.1 μM and 10 μM) at 6 h of exposure were selected based on our dose–response study (Figure 2A,B). Although there are currently no specific guidelines for thymoquinone dosage in humans, clinical studies report it to be well tolerated at daily doses of 1–5 mL of black seed oil, corresponding to roughly ≤30 mg thymoquinone per day [68]. Incorporating black seed oil as part of an antioxidant-rich diet could be a feasible preventive approach for AMD, though further clinical studies are required to determine its precise therapeutic dosage and frequency.

Previous studies showed that thymoquinone decreased oxidative stress damage through NF-κβ signalling in in vivo and in vitro AD and PD models [69,70]. In one of these studies, Velagapudi et al. (2017), showed thymoquinone was able to reduce LPS-induced oxidative stress in BV2 microglial cells by lowering the levels of TNFα, IL-6, IL and Il-1β [71]. The protective effect of thymoquinone was postulated to act through modulation of the NF-κβ signalling pathway, as measured by reductions in phosphorylated Iκβ, phosphorylated P65, the NF-kB binding capability and NF-kB luciferase activity [70]. These studies showed that thymoquinone significantly decreased NF-κβ-induced cytokines at doses between 2.5 µM and 12 µM. In addition, thymoquinone was able to reverse the stimulation of TNFα in LPS-exposed rat basophil cell lines at a 10 µM concentration [71]. Similar effects of thymoquinone attenuating the activation of TNFα were also observed rat liver [72]. This evidence supports the notion that thymoquinone exerts its protective effects by lowering the activity of the NF-κβ signalling pathway, possibly by modulating transcription of pro-inflammatory-related genes. These genes were similarly shown to be upregulated in AMD conditions [73].

Given the evidence that thymoquinone suppresses pro-inflammatory cytokines via modulation of NF-κβ signalling, subsequent studies have focused on elucidating the underlying mechanism responsible for this attenuation. Thymoquinone has been reported to influence NF-κβ activity in various cell models by decreasing the cytoplasmic P65 levels, in addition to reductions in pP65, alleviation of Iκβα degradation and inhibition of the pP65 DNA-binding capability in a concentration- and time-dependent manner [40,56,71,74,75,76]. Most studies suggest thymoquinone protection in NF-κβ signalling is achieved through a reduction in pP65 expression in the nucleus [75,76,77]. El-Gazzar et al. (2007) showed that thymoquinone modulates LPS-induced TNFα production and proved that this alteration occurs through an increase in the amount of the repressive NF-κB p50 homodimer and inhibition of P65 activation [78]. Their data also showed that the protective effect was not due to interferences in P65 DNA binding capability. In contrast, Velagapudi et al. (2017) reported that thymoquinone may act by reducing P65 DNA-binding activity, thereby limiting the release of pro-inflammatory cytokines [70]. In addition, Wang et al. (2015) and Velagapudi et al. (2017) proposed that thymoquinone-mediated NF-κβ modulation could involve inhibition of Iκβα phosphorylation, consequently preventing P65 phosphorylation [69,70].

As inflammation plays a critical role in AMD development and pathogenesis, investigating thymoquinone’s modulation of the NF-κβ pathway may shed new light, promoting understanding of the molecular mechanism of thymoquinone, and help in developing an alternative therapeutic agent. To the best of our knowledge, this is the first report investigating the effect of thymoquinone on TNFα- induced NF-κβ signalling in RPE cells. In line with Sethi et al. (2008), our current study shows that thymoquinone was able to reduce the exaggerating effect of TNFα stimulation in both non-AGE-exposed and AGE-exposed RPE cells by reducing the level of P65 (Figure 3C and Figure 4C) [40]. Significant reductions were also observed in the expression of pP65 (activated P65) (Figure 4D) and Iκβα (the inhibitor of P65) (Figure 3B and Figure 4B) at both the gene expression level and the protein expression level. Reductions in these key effectors proteins may suggest that thymoquinone protects RPE cells against TNFα-induced oxidative stress via the NF-κβ signalling pathway by suppressing the whole NFκβ signalling pathway, especially in AGE-exposed cells, thereby suppressing the release of pro-inflammatory cytokines TNFα, IL-1β and IL-6, as previously reported [70]. Although a reduction in P65 expression was observed in both non-AGE-exposed and AGE-exposed cells, the fold decrease in the AGE-exposed cells was higher with thymoquinone treatment. Thymoquinone pretreatment also produced similar reductions in Iκβα and pP65 expression, with a more pronounced effect observed in AGE-exposed cells, suggesting heightened sensitivity of these cells to the treatment. From these data, we can conclude that AGE-exposed cells are not only more vulnerable to oxidative stress stimulation, as previously reported [22], but they also are more sensitive towards antioxidant treatment.

In the current study, we are focused only on the effect of thymoquinone on the NF-κβ signalling pathway in RPE cells. In a normal cell redox state, there is a balance between the level of oxidants and antioxidants to maintain the cell’s functions and prevent cell death. However, this balance is compromised during ageing. Our study revealed that thymoquinone was able to restore the activation of the NF-κβ signalling pathway, possibly preventing inflammation. On top of this, Hu et al. (2019) have previously demonstrated that thymoquinone can protect RPE cells from H_2_O_2_-induced oxidative stress and apoptosis in the RPE via modulation of the Nrf2/HO-1 signalling pathway, a pathway responsible for regulating gene-related antioxidant properties [35]. Thymoquinone acts on this pathway by elevating nuclear localization, increasing the DNA-binding capability, increasing Nrf2 transcriptional activity and increasing heme oxygenase-1 (HO-1) protein expression and NAD(P)H: quinone oxidoreductase [70]. These genes are responsible for regulating the cellular response to oxidants. Taken together, these findings suggest that the observed suppression of NF-κβ signalling in our study may occur, at least in part, through concurrent activation of the Nrf2/HO-1 antioxidant pathway. Also from these findings, we can conclude that thymoquinone may not only act by decreasing the transcription of pro-inflammatory-related genes through NF-κβ signalling but also by increasing the levels of antioxidants through modulation of Nrf2 signalling.

While this study provides new insights into the anti-inflammatory and antioxidative effects of thymoquinone in RPE cells, several limitations should be acknowledged. Direct biochemical markers of oxidative stress (e.g., ROS, MDA/4-HNE and GSH/GSSG) and downstream cytokine release (e.g., IL-6, IL-8 and MCP-1) were not assessed due to resource constraints. However, our conclusions are supported by consistent indirect evidence, including modulation of NF-κB signalling effectors and morphological changes indicative of oxidative stress. Future studies incorporating these assays would provide stronger mechanistic validation. Additionally, some molecular analyses were performed with a limited number of biological replicates due to sample limitations, though the reproducibility of the observed trends supports the robustness of our findings. Lastly, the use of ARPE-19 cells, while suitable for initial mechanistic exploration, remains a limitation, given their metabolic and oxidative differences relative to native RPE cells. Hence, we suggest that future validation using primary or iPSC-derived RPE models will be essential to strengthen translational relevance.

Overall, these findings underscore the therapeutic potential of thymoquinone as a promising candidate for managing AMD, where oxidative stress and inflammation arise in and around the RPE layer, leading to RPE dysfunction and cell death, thereby affecting vision. Thymoquinone has been shown to reduce the expression levels of P65, pP65 and Iκβα and to potently reduce the overall activation of the NF-κβ signalling pathway, possibly reducing transcription of pro-inflammatory-related genes. Although further studies are needed to understand the downstream effect of thymoquinone on NF-κβ signalling, these results provide important mechanistic insights into the signalling underlying RPE-derived inflammatory reactions and thereby provide a theoretical basis for further development of thymoquinone usage in AMD.

## 4. Materials and Methods

### 4.1. Materials

The Human RPE cell line (ARPE-19) was purchased from the American Type Culture Collection (ATCC) (ATCC, Rockville, MD, USA). Solubilised basement membrane matrix extract, Matrigel (MG), was obtained from BD Biosciences (BD Biosciences, Oxford, UK). Thymoquinone (≥98%), Foetal Bovine Serum (FBS) and Anti Mouse IgG (whole molecule)-peroxidase antibody were acquired from Sigma Aldrich (Sigma, Dorset, UK). Dulbecco’s modified Eagle’s Medium/Nutrient Mixture F12 Ham (DMEM-F12), Trypsin/EDTA (0.05%), non-fat dry powdered milk, 3-[4,5-dimethyltiazol-2-yl]-2,5-diphenyl-tetrazolium bromide (MTT) and a Revert Aid First Strand cDNA synthesis kit were obtained from Thermo Fisher Scientific (Thermo Fischer Scientific, Carlsbad, CA, USA). An RNeasy Plus Mini extraction kit and QuantiNova SYBR Green PCR Kit were obtained from Qiagen (Qiagen, Hilden, Germany). All the antibodies—Anti P65, Anti IKB-α, Anti GAPDH and Anti Rabbit IgG (whole-molecule)-peroxidase antibodies—were obtained from Abcam (Abcam, Cambridge, UK). Anti Phospho-NF-κβ P65, Ser536 (pP65) was obtained from Cell Signalling Technology (Cell Signalling Technology, Danvers, MA, USA).

### 4.2. RPE Cell Culture and AGE Modification of Extracellular Matrix (ECM)

ARPE-19 cells were maintained in a 1:1 mixture of DMEM/F12 medium supplemented with 10% foetal bovine serum. The cells were maintained in 5% CO_2_ at 37 °C with changes of media between days 2 and 3. AGE experiments were carried out in standard 6-well plates coated with a layer of solubilised basement membrane matrix (Matrigel). The plates were coated by adding Matrigel to the well, followed by 1 h incubation at 37 °C for polymerisation. The Matrigel was used to mimic the innermost layer of Bruch’s membrane. To mimic the aged condition, the Matrigel was AGE-modified as previously described [22,79]. AGE formation on Matrigel in wells was induced through incubation with 100 m mol L^−1^ glycolaldehyde at 37 °C for 4 h. For control matrices, glycolaldehyde was replaced with PBS. Subsequently, the wells were thoroughly washed with PBS to remove traces of glycolaldehyde. The chemical reaction was terminated and further inhibited by incubating the Matrigel with 50 mmol L^−1^ sodium borohydride at 4 °C overnight, followed by thorough washing. ARPE-19 cells were seeded onto this control and AGE-treated Matrigel at a density of 1 × 10^4^ cells per well. The concentration of cells was scaled up and down depending on the plate size using this cell concentration. During the early days of culturing, the cells were fed every three days with DMEM/F12 with 10% FCS, changing to 2% FCS on the fifth day post seeding to help them form stable monolayers of cells.

### 4.3. Thymoquinone Treatment

A fresh thymoquinone stock solution was prepared by dissolving thymoquinone powder in DMSO, which served as a vehicle. The stock was further diluted to the appropriate concentration for experiments with the DMSO concentration fixed at 0.025%, which was used in control cells. The confluent cells were treated with 0.1 µM and 10 µM of thymoquinone for 6 h, after which further treatment was carried out.

### 4.4. TNFα Treatment

Following respective durations for AGE exposure and thymoquinone pretreatment, the confluent ARPE-19 cells were incubated with 10 ng/mL TNFα for 2 h. Subsequently, cells were thoroughly washed with PBS, and the lysates were harvested for downstream analysis.

### 4.5. Cell Viability Assay

Cell viability was determined using a 3-(4,5-dimethylthiazol-2-yl)-2,5-diphenyltetrazolium bromide (MTT) assay. Briefly, RPE cells were seeded into a 96-well plate at 5 × 10^4^ cells/well and cultured for 24 h to reach 65–80% confluency. After incubation, cells were subjected to appropriate treatment, and MTT was added at a final concentration of 0.5 mg/mL. The plate was incubated at 37 °C for 4 h. The supernatant was then aspirated, and DMSO was added to each well to dissolve the formed formazan. The plate was agitated for 10 min using an automated plate reader, and absorbance was measured at 570 nm using a microplate spectrophotometer. Cell viability was expressed as a percentage relative to the control group, which was set at 100%.

### 4.6. Gene Expression Analysis by Real-Time PCR

Total RNA was extracted using an RNeasy Plus Mini Kit (Qiagen, Hilden, Germany), and cDNA was synthesized using a RevertAid First Strand cDNA synthesis kit (Thermo Scientific, Waltham, MA, USA), following the manufacturer protocols. Quantitative PCR was then performed with a QuantiNova SYBR Green PCR Kit (Qiagen, Hilden, Germany) using an ABI Step-One Plus Real-Time PCR system. Each experimental condition included at least three biological replicates, and each cDNA was analysed in triplicate. Primer sequences used for the amplification are provided in Table 1. Gene expression levels were normalised to reference genes β-actin and β-tubulin, and relative expression was calculated using the efficiency-corrected ddCT method, with the calibrator sample assigned an arbitrary value of 1. Amplification specificity was verified by melt curve analysis.

### 4.7. Protein Detection by Western Blotting

Protein concentration in cell lysates was quantified using a BCA protein Assay kit (Thermo Scientific, USA). Equal amounts (in µg) of protein samples were applied to the 10% sodium dodecyl sulphate polyacrylamide electrophoresis gels, alongside a molecular weight marker. Proteins were then transferred onto a nitrocellulose membrane (VWR) and blocked with blocking buffer (5% non-fat milk) for 1 h at room temperature with gentle agitation. The membranes were subsequently washed and incubated overnight with primary antibodies (P65, IKBA, pP65 and GAPDH) at 4 °C. Following washes, the membranes were incubated with the appropriate secondary antibody (anti-rabbit or anti-goat antibody) at 37 °C for another hour. Antibody details are provided in Table 2. Protein bands were visualised using an enhanced chemiluminescent substrate kit and imaged using Geldoc instruments (Thermo Scientific, USA). Densitometry analysis was performed using Image Studio Lite Ver 5.2, and protein expression levels were normalised to the loading control (GAPDH).

### 4.8. Statistical Analysis

Data analysis was performed using Microsoft Excel commercial software (Version 2010, Microsoft UK Ltd., Reading, UK) and GraphPad Prism (Version 6.1, GraphPad Software, CA, USA). Data are presented as the mean ± standard error of the mean (SEM). Student’s *t*-test and two-way ANOVA were used, and a *p*-value < 0.05 was considered to be statistically significant.

## Figures and Tables

**Table 1:** List of primers used for gene expression analysis.

Primer	Template	5′→3′ Sequence
Iĸβα	Forward	GAAGTGATCCGCCAGGTGAA
	Reverse	CTCACAGGCAAGGTGTAGGG
p65	Forward	CCAGACCAACAACAACCCCT
	Reverse	TCACTCGGCAGATCTTGAGC
β- actin	Forward	CACCATTGGCAATGAGCGGTTC
	Reverse	AGGTCTTTGCGGATGTCCACGT
β- tubulin	Forward	CTGGACCGCATCTCTGTGTACT
	Reverse	GCCAAAAGGACCTGAGCGAACA

**Table 2:** Antibodies used for protein expression analysis.

Antibody	Dilution
Anti NF-kB P65	1:500
Anti Phospho-NF-kB P65 (Ser536)	1:500
Anti IKB-α	1:500
Anti GAPDH	1:10,000
Secondary horseradish peroxidase (HRP)-conjugated anti-rabbit	1:1000
Secondary horseradish peroxidase (HRP)- conjugated anti-mouse	1:2000

## Data Availability

The original contributions presented in this study are included in the article. Further inquiries can be directed to the corresponding author.

## Supplementary Materials

The following supporting information can be downloaded at: https://www.mdpi.com/article/10.3390/ijms262311473/s1.

## Abbreviations

The following abbreviations are used in this manuscript:

AD	Alzheimer Disease
AGEs	Advanced Glycation Endproducts
AMD	Age-related Macular Degeneration
MTT	3-(4,5-dimethylthiazol-2-yl)-2,5-diphenyltetrazolium bromide
PD	Parkinson Disease
RPE	Retinal Pigment Epithelium
SEM	Standard Error of the Mean
