# Correction: Mahmud et al. Thymoquinone Attenuates NF-κβ Signalling Activation in Retinal Pigment Epithelium Cells Under AMD-Mimicking Conditions. *Int. J. Mol. Sci.* 2025, *26*, 11473

**DOI:** 10.3390/ijms27125500

**Published:** 2026-06-18

**Authors:** Nur Musfirah Mahmud, Luminita Paraoan, Tengku Ain Kamalden

**Affiliations:** 1 UM Eye Research Centre, Department of Ophthalmology, Faculty of Medicine, Universiti Malaya, Kuala Lumpur 50603, Malaysia; musfirah@um.edu.my; 2 Department of Life Sciences, Faculty of Science and Engineering, Manchester Metropolitan University, Chester Street, Manchester M1 5GD, UK; l.paraoan@mmu.ac.uk

The following reference [59] has been retracted and has to be removed from the original publication [1].

Ref. [59]. Abo Mansour, H.E.; Elberri, A.I.; Ghoneim, M.E.-S.; Samman, W.A.; Alhaddad, A.A.; Abdallah, M.S.; El-Berri, E.I.; Salem, M.A.; Mosalam, E.M. The Potential Neuroprotective Effect of Thymoquinone on Scopolamine-Induced In Vivo Alzheimer’s Disease-like Condition: Mechanistic Insights. *Molecules* **2023**, *28*, 6566. Retraction in *Molecules* **2025**, *30*, 4436. https://doi.org/10.3390/molecules30224436.

The scientific content of [59] was not replaced with a new citation, as the information it supported is already adequately covered by the existing references. Therefore, its removal does not affect the content, interpretation, or conclusions of the manuscript. 

The citation for Tavakkoli et al., 2017 was correctly included as Reference [68]. However, due to an EndNote synchronization issue, the same reference was inadvertently duplicated and appeared again as Reference [79]. After updating and refreshing the EndNote library, the duplicate entry at Reference [79] was removed. 

Ref. [68]. Tavakkoli, A.; Mahdian, V.; Razavi, B.M.; Hosseinzadeh, H. Review on Clinical Trials of Black Seed (Nigella sativa) and Its Active Constituent, Thymoquinone. *J. Pharmacopunct.* **2017**, *20*, 179–193. 

Upon further review, Glenn et al., 2009 had been cited in the manuscript text but was inadvertently retained in APA format rather than being properly linked through EndNote. As a result, the reference was not generated in the reference list in the previous version of the manuscript. After updating the EndNote library and refreshing the bibliography, this issue was resolved, and Glenn et al., 2009 is now correctly included as Reference [79]. Consequently, although Reference [59] was removed, the total number of references remains 79, as the previously omitted Glenn et al., 2009 reference has now been correctly restored to the reference list. 

Ref. [79] Glenn, J.V.; Mahaffy, H.; Wu, K.; Smith, G.; Nagai, R.; Simpson, D.A.; Boulton, M.E.; Stitt, A.W. Advanced glycation end product (AGE) accumulation on Bruch’s membrane: Links to age-related RPE dysfunction. *Investig. Ophthalmol. Vis. Sci.* **2009**, *50*, 441–451.

With this correction, the order of some references has been adjusted accordingly. The authors state that the scientific conclusions are unaffected. This correction was approved by the Academic Editor. The original publication has been updated. 
