# Supplementary material for: Thymoquinone Attenuates NF-κβ Signalling Activation in Retinal Pigment Epithelium Cells Under AMD-Mimicking Conditions
Source: Int J Mol Sci. 2025 Nov 27;26(23):11473. doi: 10.3390/ijms262311473 (PMC12692087; doi:10.3390/ijms262311473)
Supplement: Supplementary file 1 [file ijms-26-11473-s001.zip › ijms-3882194-supplementary-update.docx]

Supplementary Table S1

**Table S1.** Data for Figure 2B

| Concentration (µM) | Cell Viability (%) | | |
| --- | --- | --- | --- |
|  | 2h (Mean ± SEM) | 6h (Mean ± SEM) | 24h (Mean ± SEM) |
| 0 | 100 ± 0.0 | 100 ± 0.0 | 100 ± 0.0 |
| 0.1 | 96.8 ± 1.2 | 104.7 ± 5.9 | 94.8 ± 5.5 |
| 1 | 93.9 ± 7.0 | 102.8 ± 8.2 | 97.5 ± 8.6 |
| 5 | 86.9 ± 4.4 | 97.1 ± 11.4 | 86.9 ± 6.3 |
| 10 | 88.9 ± 11.3 | 91.2 ± 8.2 | 89.0 ± 7.3 |
| 50 | 56.8 ± 13.6 | 52.7 ± 12.9 | 31.5 ± 4.3 |
| 75 | 45.9 ± 6.4 | 35.5 ± 5.4 | 12.4 ± 1.8 |
| 100 | 42.0 ± 8.1 | 32.3 ± 4.7 | 9.00 ± 0.8 |

**Table S2.** Data for Figure 2C

|  | Cell Viability (%) | | | |
| --- | --- | --- | --- | --- |
| Group | Control | TNFα | TQ1 (0.1µM) + TNFα | TQ2 (10µM) + TNFα |
| Mean ± SEM | 100 ± 0.0 | 109.5 ± 8.0 | 93.0 ± 6.0 | 92.8 ± 4.7 |

**Table S3.** Data for Figure 3B

| Group | | Mean ± SEM |
| --- | --- | --- |
| Non - AGEs | Control | 1.0 ± 0.0 |
|  | TNFα | 3.4 ± 1.2 |
|  | TQ1 (0.1µM) + TNFα | 1.6 ± 0.5 |
|  | TQ2 (10µM) + TNFα | 1.5 ± 0.3 |
| AGEs | Control | 1.0 ± 0.0 |
|  | TNFα | 3.3 ± 0.7 |
|  | TQ1 (0.1µM) + TNFα | 1.4 ± 0.4 |
|  | TQ2 (10µM) + TNFα | 0.9 ± 0.4 |

**Table S4.** Data for Figure 3C

| Group | | Mean ± SEM |
| --- | --- | --- |
| Non - AGEs | Control | 1.0 ± 0.0 |
|  | TNFα | 2.9 ± 0.6 |
|  | TQ1 (0.1µM) + TNFα | 2.3 ± 0.9 |
|  | TQ2 (10µM) + TNFα | 2.2 ± 0.9 |
| AGEs | Control | 1.0 ± 0.0 |
|  | TNFα | 3.8 ± 0.4 |
|  | TQ1 (0.1µM) + TNFα | 2.4 ± 0.3 |
|  | TQ2 (10µM) + TNFα | 2.4 ± 0.3 |

**Table S5.** Data for Figure 4B

| Group | | Mean ± SEM |
| --- | --- | --- |
| Non - AGEs | Control | 1.0 ± 0.0 |
|  | TNFα | 2.8 ± 1.1 |
|  | TQ1 (0.1µM) + TNFα | 1.8 ± 0.6 |
|  | TQ2 (10µM) + TNFα | 1.8 ± 0.9 |
| AGEs | Control | 1.0 ± 0.0 |
|  | TNFα | 4.0 ± 0.7 |
|  | TQ1 (0.1µM) + TNFα | 2.5 ± 0.5 |
|  | TQ2 (10µM) + TNFα | 2.7 ± 0.6 |

**Table S6.** Data for Figure 4C

| Group | | Mean ± SEM |
| --- | --- | --- |
| Non - AGEs | Control | 1.0 ± 0.0 |
|  | TNFα | 1.9 ± 0.3 |
|  | TQ1 (0.1µM) + TNFα | 1.6 ± 0.5 |
|  | TQ2 (10µM) + TNFα | 1.4 ± 0.5 |
| AGEs | Control | 1.0 ± 0.0 |
|  | TNFα | 2.1 ± 0.3 |
|  | TQ1 (0.1µM) + TNFα | 1.4 ± 0.3 |
|  | TQ2 (10µM) + TNFα | 1.3 ± 0.2 |

**Table S7.** Data for Figure 4D

| Group | | Mean ± SEM |
| --- | --- | --- |
| Non - AGEs | Control | 1.0 ± 0.0 |
|  | TNFα | 2.6 ± 0.2 |
|  | TQ1 (0.1µM) + TNFα | 1.8 ± 0.7 |
|  | TQ2 (10µM) + TNFα | 1.7 ± 0.7 |
| AGEs | Control | 1.0 ± 0.0 |
|  | TNFα | 2.8 ± 0.3 |
|  | TQ1 (0.1µM) + TNFα | 1.7 ± 0.5 |
|  | TQ2 (10µM) + TNFα | 1.8 ± 1.0 |
